# EXercise to prevent AnthrCycline-based Cardio-Toxicity (EXACT) in individuals with breast or hematological cancers: a feasibility study protocol

**DOI:** 10.1186/s40814-016-0084-9

**Published:** 2016-08-05

**Authors:** Melanie R. Keats, Scott A. Grandy, Nicholas Giacomantonio, David MacDonald, Miroslaw Rajda, Tallal Younis

**Affiliations:** 1 School of Health and Human Performance (Kinesiology), Dalhousie University, PO Box 15000, 6230 South Street, Halifax, Nova Scotia B3H 4R2 Canada; 2 Division of Cardiology, Department of Medicine, Dalhousie University, Halifax, Nova Scotia Canada; 3 Division of Hematology, Department of Medicine, Dalhousie University, Halifax, Nova Scotia Canada; 4 Division of Medical Oncology, Department of Medicine, Dalhousie University, Halifax, Nova Scotia Canada; 5 QEII Health Sciences Center—HI Site, Suite 2261—1796 Summer St., Halifax, Nova Scotia B3H 3A6 Canada; 6 QEII Health Sciences Center, 1276 South Part Street, Halifax, Nova Scotia B3H 2Y9 Canada

**Keywords:** Feasibility study, Breast cancer, Hematological cancer, Aerobic exercise training, Cardiotoxicity, Anthracyclines

## Abstract

**Background:**

Anthracyclines (AC), widely used and effective anticancer agents, are known to induce both acute and chronic declines in cardiovascular health, ranging in severity from asymptomatic, subclinical dysfunction to substantial cardiomyopathy leading to congestive heart failure and death. There is substantial evidence that physical activity, higher levels of cardiorespiratory fitness, and exercise therapy can help prevent cardiovascular disease. Moreover, animal studies have shown that exercise performed concomitantly with AC treatment may attenuate early cardiac damage that results from AC exposure. Our primary objective is to assess the feasibility of a 12-week aerobic exercise training (AET) program in patients receiving AC-based chemotherapy.

**Methods/design:**

This is a prospective, single-arm (pre-post-test design), feasibility study of a supervised 12-week progressive, light-to-moderate to moderate-to-vigorous intensity AET program for patients (18–65 years) receiving AC chemotherapeutic treatment for a primary/non-recurrent breast cancer or hematological malignancy. Both feasibility (e.g., participant recruitment, program adherence, safety) and intervention outcome (e.g., biological markers of cardiotoxicity, aerobic capacity, quality of life) measures will be collected. The AET program will include two, 45-min community-based exercise sessions (treadmill or cycle) per week for a total of 12 weeks. All exercise sessions will be supervised by trained exercise specialists.

**Discussion:**

Data from the EXACT study will be evaluated to determine the need to refine patient recruitment methods and general acceptability of the AET program. Preliminary data on the effects of the AET intervention on pertinent cardiac and health outcomes will also be evaluated and used to inform future studies in terms of the most appropriate outcome measure(s) to adopt and sample size estimation.

**Trial registration:**

ClinicalTrails.gov, NCT02471053

## Background

As the numbers of cancer survivors grow, the long-term adverse effects of cancer therapy are becoming increasingly apparent. Perhaps the most prominent of these are the toxic effects on the heart (cardiotoxicity) which may lead to cardiac dysfunction and increased risk of cardiovascular disease (CVD) [1]. Of note, data indicate that the magnitude of CVD risk for long-term survivors may exceed the risk of recurrent cancer and represents the leading non-cancer cause of death among survivors of breast cancer and Hodgkin’s disease [2–5]. Thus, it is clear that the long-term cardiotoxic effects of cancer therapy represent a significant concern for many cancer survivors.

While the advent of modern cancer therapies has translated into substantially improved survival, several widely used therapies have been shown to increase the risk of cardiac toxicity. Cancer therapeutics have been associated with a number of long-term cardiac complications such as decreased left ventricular function, hypertension, arrhythmias, cardiac ischemia, pericardial and valvular disease, cardiomyopathy, and congestive heart failure [6, 7]. For example, anthracyclines (AC), a chemotherapeutic agent commonly utilized in the management of many adult cancers including breast and hematological malignancies, are known to be cardiotoxic. The risk of cardiotoxicity associated with AC impacts its benefit/risk profile and ultimately limits the drugs clinical utility [6–10]. The adverse effects of AC can be associated with acute, early-onset, and chronic cardiomyocyte injury that can range in severity from asymptomatic subclinical left ventricular dysfunction to significant cardiac impairment and symptomatic heart failure [9]. Although several factors underlie the cardiotoxic effects of AC on the heart, the primary causative factor underlying acute cardiotoxicity appears to involve an inflammatory response while the formation of free radicals appears to be related to chronic AC cardiotoxicity [8] both of which can contribute to the development and progression of CVD [11–13].

The risk of developing cardiotoxicity appears to be predominately related to the total cumulative AC dose. Although the incidence of AC-induced cardiotoxicity appears to be greatest at doxorubicin dosages exceeding 700 mg/m^2^ [6, 7], cardiotoxicity has been reported in patients who have received doses as low as 100 mg/m^2^, suggesting that there is no reliably “safe” dose [14]. Specifically, the risk of heart failure has been reported in 3 to 5 % of patients that receive a cumulative dose of 400 mg/m^2^. As cumulative dose increases, so too does the incidence of heart failure. For example, at cumulative doses of 550 and 700 mg/m^2^, the incidence of heart failure has been reported as 7–26 and 18–48 %, respectively [15]. Furthermore, it has been reported that the cumulative incidence of heart failure in elderly breast cancer patients 10 years post-AC-based treatment was 38 % [15].

Patients with a pre-existing history of hypertension or CVD, those who receive AC concurrently with other known cardiotoxic agents (e.g., trastuzumab), advanced age, female gender, or those treated with mediastinal radiation are at a greater risk of developing AC-mediated CVD [8, 14]. Lifestyle factors (e.g., physical inactivity, smoking, high BMI) may also contribute to the increased risk of CVD in this population [16].

While AC cardiotoxicity is thought to cause irreversible cardiomyocyte damage (i.e., type I), recent data suggests that early detection and intervention may mitigate or reverse myocardial cell injury. Cardinale and colleagues [17] prospectively evaluated the left ventricular ejection fraction (LVEF) in 2625 AC-treated breast and non-Hodgkin lymphoma patients before, during, and after chemotherapy for a median follow-up of 5.2 years. They found that cardiotoxicity (defined as a >10 % reduction in LVEF from baseline and an end LVEF of <50 %) occurred in 9 % of patients. The majority of these cases (98 %) emerged within the first year of completing AC therapy (median time to development of cardiotoxicity was 3.5 months). Upon identification of cardiotoxicity, early heart failure therapy was initiated and full (11 %) or partial (71 %) recovery was reported in 82 % of the cases. Although this study provides data to support early detection and treatment of cardiotoxicity, challenges remain. Groarke and Nohria [18] note that the early identification of cardiotoxicity often involves the reconsideration of chemotherapy protocols resulting in either dose reductions or treatment substitutions. Given that 97 % of the reported deaths in the Cardinale et al. [17] study were cancer related, the balance of minimizing the risk of cardiotoxicity against antineoplastic efficacy becomes a significant and ongoing concern. Accordingly, intervention strategies that complement existing antineoplastic treatment strategies while decreasing the risk of cardiotoxicity are needed.

Physical activity and exercise has long been established as a powerful tool in the prevention and treatment of chronic diseases, including some cancers and CVD [19, 20]. Exercise has also been shown to reduce all-cause mortality as well as both cancer [21] and cardiovascular-related mortality [22]. Importantly, while exercise can increase aerobic fitness as well as modify traditional CVD risk factors [23], exercise may also help protect the heart from the associated cardiotoxic effects of AC-based therapy [24]. For example, several animal studies have shown that regular exercise performed prior to or concomitantly with AC treatment helps to preserve cardiac function [25–30]. Pre-treatment exercise has also been shown to decrease AC accumulation in the heart, which helps to decrease cardiotoxicity [31]. Furthermore, studies of AC-mediated cardiotoxicity have shown that regular exercise can increase antioxidant defenses [29, 32] and decrease inflammatory markers [33, 34]. These changes decrease the impact of AC-mediated oxidative stress and inflammation, thus improving myocardiocyte survival [25, 35, 36]. Thus, while much is left to be explored, exercise may prove effective in the management of AC-mediated cardiotoxicity as well as improving overall cancer survival.

Despite the increased risk of cardiotoxicity and the potential benefits of exercise therapy, the efficacy of exercise as a therapeutic intervention for the prevention of AC-mediated cardiotoxicity remains speculative. Accordingly, the purpose of this study is to examine the feasibility and potential efficacy of a 12-week, individualized aerobic exercise training (AET) program to mitigate cardiac toxicity and patient outcomes associated with AC-based chemotherapy.

The specific objectives of the study are as follows: (1) to examine feasibility outcomes (e.g., recruitment, program adherence, and safety) and (2) to explore the effects of the exercise program on biological markers of AC-mediated cardiotoxicity (e.g., levels of inflammation) as well as patient-centered outcomes (e.g., increased aerobic capacity, quality of life). We hypothesize that an individualized AET program for cancer patients receiving active AC-based treatment will be both safe and feasible with an anticipated adherence of 66–85 % [37]. We further hypothesize that the AET program will result in improvements in the overall levels of physical activity, aerobic fitness, and quality of life. Given the dearth of knowledge surrounding both the measurement of cardiac biomarkers and the cardioprotective nature of aerobic exercise in human models, no a priori hypotheses have been made. Instead, this study will serve to inform future iterations.

## Methods/design

### Study design and procedures

**Fig. 1:**
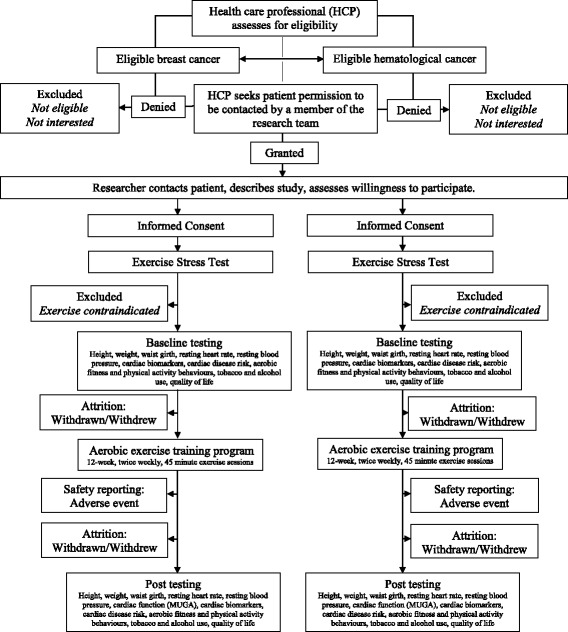
Participant flow through the EXACT study representing each cancer site separately

The protocol was drafted in accordance with the SPIRIT 2013 statement [38]. This is a single-arm, prospective (pre-post) feasibility study of a supervised 12-week light-to-moderate to moderate-to-vigorous intensity AET program. The study flow is presented for each separate cancer site in Fig. 1. Potential participants will be identified and screened for preliminary eligibility by their medical oncologist/hematologist during a regular office visit at the Queen Elizabeth II Health Sciences Center (QEII HSC) in Halifax, Nova Scotia, Canada. Pending preliminary screening for study eligibility and medical approval to participate, potential participants will be approached by a member of the medical staff to seek permission to refer them to the research coordinator to discuss the details of the study. Pending approval to be contacted by the research team, eligible participants will be provided with a detailed review of the study and informed consent will be sought by the research coordinator. Eligible participants who provide informed consent will undergo additional cardiovascular risk and fitness assessment (i.e., cardiopulmonary exercise test (CPET)) to determine study suitability and ability to safely participate in a low-to-moderate or moderate-to-vigorous aerobic training program. Those meeting the study criteria will then complete a pre-exercise baseline assessment (week 0) of anthropometric measures, select cardiac biomarkers, lifestyle behaviors, and quality of life. Based on the findings of the exercise stress test and baseline assessments, an individualized, bi-weekly AET program will be developed (weeks 1–12). Pending the completion of the 12-week training program, participants will repeat the exercise stress and cardiac function tests and the assessments measured at baseline (week 13). The study is registered with ClinicalTrails.gov (NCT02471053).

### Setting and participants

The EXercise to prevent AnthrCycline-based Cardio-Toxicity (EXACT) feasibility study will seek to recruit 20 participants receiving active AC-based chemotherapy for either a primary breast or hematological cancer at the QEII HSC. All study assessments and exercise sessions will take place at an affiliated center located in close proximity to the cancer clinics. Potential participants will be identified based on the following inclusion criteria: (1) participants are between the ages of 18 and 65 at the time of study initiation; (2) are within 8 weeks receiving the first dose of an AC-based chemotherapeutic treatment for a primary, non-metastatic, HER2-negative breast cancer, or hematological malignancy; (3) are scheduled to receive a minimum dose of 100 mg/m^2^ of doxorubicin (or equivalent) [39]; (4) have undergone a pre-treatment cardiac function test; (5) are willing to participate in a twice-weekly, 12-week community-based AET program; and (6) have medical consent from their medical oncologist/hematologist to participate in an exercise study. Any patients that meet the inclusion criteria, but have (1) significant cognitive limitations; (2) significant cardiovascular disease (i.e., myocardial infarction, cerebrovascular disease, peripheral vascular disease, congestive heart failure, or cardiomyopathy) or any known contraindication to exercise; (3) previous history of cancer; or (4) known bone metastases will be excluded from the study.

### Intervention

**Fig. 2:**
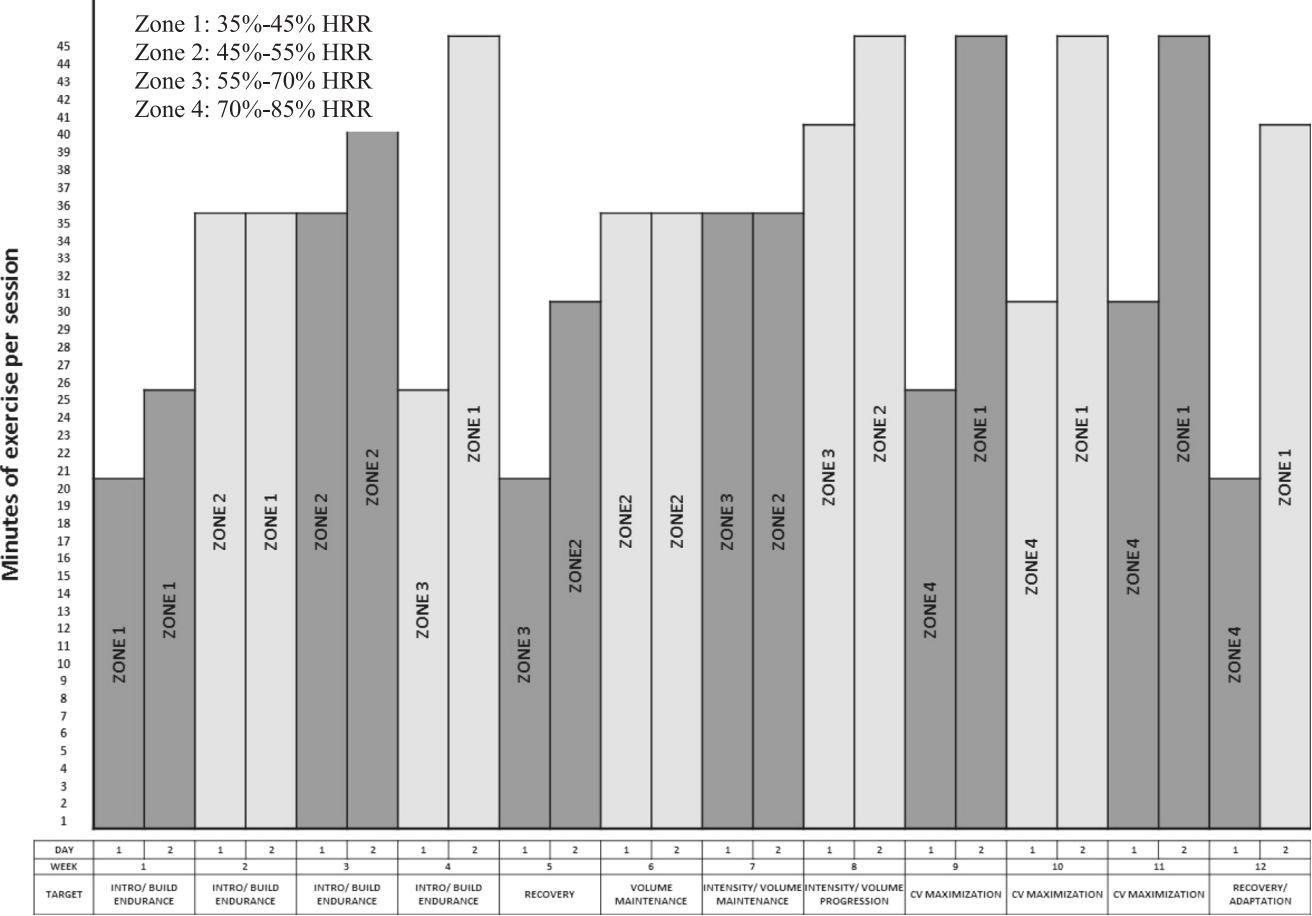
Twelve-week aerobic exercise training program [37]. Note: heart rate reserve (HRR) = % of target intensity (HR_max_ − HR_rest_) + HR_rest_

In addition to standard oncologic care, all participants will be enrolled in a 12-week AET program to commence following baseline testing. Participants will be asked to complete two supervised exercise sessions on non-consecutive days each week. Session intensity will range from light-to-moderate (as defined as 35 to 55 % heart rate reserve (HRR)) or moderate-to-vigorous activity (defined as 55 to 85 % HRR). Participants will use a wrist-worn heart rate monitor (Polar Electro Canada Inc., QC) to ensure that they are training within the appropriate target heart rate zone. Exercise sessions will begin with a group warm-up activity (5 min), followed by 20–45 min of individually tailored aerobic activity (based on the maximum heart rate achieved in the absence of any clinical symptoms during the baseline CPET) and ending with a cool down (10 min). Aerobic activities will include walking on a treadmill and/or cycling on an upright or recumbent cycle ergometer. A nonlinear approach will be used to optimize adaptations to the exercise stimulus, and session intensity will be inversely related to session duration (Fig. 2) [37]. We have elected to adopt a nonlinear exercise prescription based on the foundational principles of exercise physiology, individualization, specificity, progressive overload, and recovery [37, 40, 41]. Baseline physiological testing of the target system will be measured using an incremental CPET [42]. The results of the CPET, peak workload, and peak exercise heart rate will allow for an individualized exercise prescription, rather than a generic exercise prescription based on age-predicated maximum heart rate which can result in under- or overprescription of exercise for the participant [37]. With regards to specificity, aerobic exercise was chosen to elicit cardiovascular physiological adaptations, as the purpose of the study centers around cardiotoxicity. Progressive overload will be used to guide the pattern of exercise intensity and duration over the 12-week program [40, 41]. The varied intensity between training sessions will be used to continually perturb the cardiovascular system in order to create a physiological response. However, caution will be exercised to ensure that overtraining does not occur as this could result in excessive fatigue and/or injury [37]. Thus, the exercise protocol has been designed with periods of progressive overload (either higher intensity or longer duration) with built-in periods of rest (days off) and active recovery (lower intensity, shorter duration). This type of exercise intervention has been previously utilized in oncology settings and has shown that there are favorable adherence rates (66 to 85 % adherence) and a low incidence of adverse events [37].

To achieve a total of 150 min of light-to-moderate or moderate-to-vigorous activity each week, participants will be asked to engage in additional home-based aerobic activities (e.g., walking). Participants will be asked to keep an activity diary including a record of their resting heart rate, type of exercise performed, duration of the exercise, and maximum heart rate during all supervised and home-based exercise sessions.

### Strategies to enhance participant recruitment, adherence, and retention

Efforts to optimize participant engagement will include on-site, face-to-face recruitment and physician endorsement. Several strategies to improve participant adherence and retention will be also be employed including group-based, individually tailored exercise training sessions; personalized performance feedback (e.g., weekly activity diary review); follow-up phone calls to inquire about missed sessions; ability to make-up missed session(s) over the course of the 12-week intervention; and scheduling flexibility.

### Primary outcomes

#### Participant accrual, adherence, retention, and safety

The primary feasibility outcome measures for this study will include variables associated with participant recruitment, adherence, retention, and safety. Recruitment rate will be calculated by the number of patients who consent to participate divided by the number of deemed eligible to participate by their primary care oncologist/hematologist. Program adherence will be calculated by counting the actual number of sessions that the participant engaged in divided by the recommended number of weekly sessions. Participants will also be asked to record their activity duration (time) and intensity (as measured by heart rate) following each training session in order to calculate exercise volume. Participant retention will be determined by reporting the number of participants who complete baseline and post-intervention testing. Program safety will be assessed by weekly tracking and monitoring the number of adverse events or injuries that occur during the duration of the study. An investigators meeting will be held every 3 months to review and discuss all reported minor adverse events (e.g., muscle stiffness, activity-related fatigue) and injuries (e.g., muscle strain) for early risk identification and modification of study procedures as needed. All serious adverse events will be immediately disseminated to all study investigators for review and reported to the Nova Scotia Health Authority Research Ethics Board. There are currently no stopping guidelines for this trial.

### Secondary outcomes

Baseline demographics (age, sex, ethnicity, education, employment status, family income), lifestyle behaviors (general health, aerobic fitness, physical activity behaviors, sleep habits, tobacco use, and alcohol consumption), and medical history (cancer diagnosis, cancer treatment(s) received, comorbid health conditions, markers of cardiac risk [43]) will be collected by a combination of self-report and participant-consented medical record review.

#### Cardiac function

Resting baseline and post intervention LVEF will be performed on all participants with a multigated acquisition (MUGA) scan. MUGA is a non-invasive, cost-effective, and reliable measure of heart function by tracking blood flow through the ventricles. Serial monitoring will permit for the early detection of decreased left ventricular function [44].

#### Cardiorespiratory fitness

CPETs will be performed to determine peak aerobic capacity (VO_2_ peak) that the participant reaches in the absence of any clinical symptoms (e.g., fatigue, leg pain, changes in ECG). The CPETs will be performed in accordance with the American Heart Association (AHA) guidelines [42]. In brief, all participants will complete either the BRUCE treadmill protocol or a RAMP treadmill protocol [42]. A standard 12-lead ECG will be recorded concurrently with the CPET [42]. Resting ECG and post-exercise ECG will also be recorded. Blood pressure will be recorded manually at rest prior to the test, during the CPET (at the end of every exercise stage), and during recovery [40]. All tests will be performed by a CPET technician and supervised by a cardiologist. Upon completion, all CPET results will be reviewed by a cardiologist to determine if there is any evidence of undiagnosed pre-existing cardiovascular disease or any contraindications to participating in the study exercise protocol.

In general, maximal symptom-limited exercise testing (CPET) is a reasonably safe procedure [45]. The American Thoracic Society/American College of Chest Physicians report that the number of life-threatening complications or deaths was approximately 2 to 5 per 100,000 tests [45]. Statistics, based on a review of the literature, published by the AHA report the number of sudden cardiac deaths which ranges from 0 to 5 per 100,000 tests [46]. Similarly, systematic reviews suggest that CPET is safe for cancer patients. For example, a review of oncology studies that utilized exercise testing reports that adverse events were reported in less than 15 % of the studies and that there were no reports of death [47]. Another systematic review reported that adverse events occurred in only 1 % of oncology patients that performed exercise testing [48]. Together, the evidence clearly indicates that CPETs can be safely administered to individuals receiving and/or recovering from cancer treatment.

#### Cardiac biomarkers

Blood samples will be collected from the participants prior to and immediately following the 12-week exercise intervention to determine the participant’s lipid profile and fasting glucose. Markers of cardiac injury including high-sensitivity troponin (hs-TNT), N-terminal pro-brain natriuretic peptide (NTproBNP), and C-reactive protein (CRP) levels as well as various systemic markers of inflammation will also be collected. These tests will occur within 7 days of starting and ending the program. Samples will be processed at the QEII HSC to quantify levels of lipids, fasting glucose, hs-TNT, NTproBNP, and CRP. Serum also will be extracted from the blood samples in order to quantify markers of systemic inflammation (cytokines). A BioPlex Pro human cytokine immunoassay will be used to quantify the levels of 27 different cytokines (e.g., IL-1α, IL-1β, IL-4, IL-6, IL-10, IL-17, TNFα). Assays will be performed as per manufacturer’s instructions. A MAGPIX Suspension Array System will be used to analyze the cytokine assay. All serum samples will be stored at −80 °C until they are required for analysis. With participant consent, blood samples will be stored for use for future related studies.

#### Total physical activity

To capture additional activity behaviors, total physical activity across four life domains (i.e., job-related, house and yard work, recreation, and active transportation) will be assessed using the long version of the International Physical Activity Questionnaire (IPAQ) pre- and post-intervention. Physical activity levels as well as continuous values of MET-minutes per week (MET = metabolic equivalent of task) will be calculated according to the IPAQ scoring protocol [49, 50].

Self-reported time spent sitting (sedentary behaviors) will also be assessed by the IPAQ by calculating the sum of the time spent sitting (e.g., at a desk, watching television, reading, and time spent sitting in a vehicle for the purpose of transportation). Time spent sitting will be calculated according to the IPAQ scoring protocol [50]. The IPAQ tool has demonstrated acceptable reliability and validity in adults 18–65 years of age and is comparable to other self-reported measures of physical activity [49].

#### Anthropometrics

Body weight will be assessed to the nearest 0.1 kg, and height will be measured to the nearest 0.1 cm using a Tanita body composition analyzer. Body mass index will be calculated as the weight in kilograms divided by the height in meters squared (kg/m^2^). Waist girth measurements will be taken clear of participant clothing at the top of the iliac crest to the nearest 0.1 cm [41].

#### Patient-reported outcomes

Cancer-related fatigue and disease-specific quality of life will be assessed pre- and post-intervention using cancer-specific, self-administered Functional Assessment of Cancer Therapy (FACT) tools [51–55]. The FACT tools are reliable and valid measures of disease-specific quality of life and fatigue [51–55].

### Sample size

For this feasibility study, we will aim to recruit 20 breast and hematological cancer patients over a course of 12 months. Preliminary data will inform power analyses for a larger randomized trial. Likewise, the number of patients that we are able to recruit during this period will determine the number of collaborating centers needed to obtain sufficient participant recruitment for future studies.

#### Project management

The conduct and management of the feasibility study will be the responsibility of the research group comprised of the study investigators and research support staff. The research coordinator will be responsible for the day-to-day management of the trial including participant recruitment and retention, assisting with outcome assessments, design and conduct of the exercise intervention, and data management. The research coordinator and principal investigators (MK and SG) will meet weekly to discuss participant recruitment, outcome assessments, intervention delivery, participant safety, and data management. Additional meetings with medical co-investigators will be held every 3 months or more frequently as needed.

### Ethical governance and dissemination

This study has received ethical approval from the Nova Scotia Health Authority Research Ethics Board (REB file: NSHA ROMEO File #: 1019999). Any protocol amendments will be submitted for further review and approval. Study findings will be presented at conferences and submitted for publication in peer-reviewed journals. Only those team members who fulfill authorship criteria will be included as authors on any future publications. Professional writers will not be employed.

### Confidentiality and access to data

All study personnel will be trained in the requirement of participants’ confidentiality according to the Tri-Council Policy Statement: Ethical Conduct for Research Involving Humans. Participant identification numbers will be assigned upon study enrollment, and access to personally identifiable information will be limited to members of the research team. All data files will be stored on a password-protected computer in a secure research laboratory.

#### Data management and analysis

De-identified data will be double entered in SPSS version 22, and basic descriptive statistics will be conducted to describe the study sample and outcomes. Rates for process measures (e.g., recruitment, adherence) will be reported. Exploratory correlational analyses will also be conducted to examine the relationship between program adherence and secondary outcome measures. Reasons for non-participation and attrition will be recorded to inform future recruitment and retention strategies. Program safety will be determined by examining the total number adverse events that occur over the duration of the 12-week exercise program. The total number of adverse events over the course of the study will be divided by the total number of participant hours to determine the number of adverse events per participant hour. Secondary outcome measures will be assessed at baseline (week 0) and post-intervention (week 13) using descriptive statistics and 95 % confidence interval estimation. Change between time points will also be assessed in this way, and within-group correlations will be calculated between the two time points. The data will be used to determine the most appropriate outcome measure for the main study and in sample size calculations required for a future grant submission. Descriptive statistics and confidence intervals will also be used to examine the trends in the biomarker data. This information will be used to determine which markers to focus on in future grant submissions. Should we find that the biomarker data is normally distributed, then a 95 % confidence interval for paired normally distributed data will be used; otherwise, a non-parametric approach will be taken.

## Discussion

Given the established antineoplastic effectiveness of AC-based therapies, non-competing strategies to prevent or decrease the risk of AC-mediated cardiotoxicity and cardiovascular complications are needed to improve survivorship outcomes. Despite initial promising findings, the role of exercise in the management of AC-mediated cardiotoxicity remains to be fully elucidated. The primary objective of the EXACT study is to shed light on the feasibility of a supervised, progressive aerobic exercise program for patients undergoing active cancer treatment. We will also attempt to elucidate how exercise may provide incremental benefit with respect to the prevention of AC-mediated cardiotoxicity.

On the whole, this study represents an important first step towards designing large-scale studies to understand how regular aerobic exercise impacts heart health in patients undergoing active AC treatment. This feasibility study will provide additional insight into the practical barriers to exercise in patients undergoing active chemotherapy and will allow for the development of strategies to improve the acceptance and adherence to appropriate regimes. Given the relatively small, geographically dispersed population in Nova Scotia, it is likely that there will be a need for multi-center trials that include a home and/or community-based component to maximize participant adherence. Findings from this study will inform the safety, design, and systematic conduct of such future trials. Furthermore, the exploration of non-invasive and safe screening outcome measures (i.e., cardiac biomarkers, LVEF, exercise stress testing) that underlie the potential cardioprotective nature of aerobic exercise will serve to better elucidate the mechanisms of benefit and inform future studies [56]. This will subsequently improve the proper measure and interpretation of the effectiveness of exercise training in maximizing cardiovascular health and to minimize cardiotoxicity of the AC-based chemotherapeutic regimes. Ultimately, it is our goal to be able to identify patients and survivors at the greatest risk of adverse cardiovascular events, evaluate the effects of exercise to ameliorate this risk, and foster change in the current standards of care to reflect improved patient quality of life and clinical outcomes.

### Trial status

ClinicalTrials.gov identifier NCT02471053 (protocol version 1.0; 27 May 2015). Recruitment for the study commenced in February 2016.

## Abbreviations

AET, aerobic exercise training; AHA, American Heart Association; AC, anthracyclines; CPET, cardiopulmonary exercise stress test; CVD, cardiovascular disease; CRP, C-reactive protein; FACT, Functional Assessment of Cancer Therapy; HRR, heart rate reserve; hs-TNT, high-sensitivity troponin; IPAQ, International Physical Activity Questionnaire; LVEF, left ventricular ejection fraction; NTproBNP, N-terminal pro-brain natriuretic peptide
